# Viewpoint: Investing in strategies to support mental health recovery from the COVID-19 pandemic

**DOI:** 10.1192/j.eurpsy.2021.28

**Published:** 2021-04-26

**Authors:** David McDaid

**Affiliations:** Care Policy and Evaluation Centre, Department of Health Policy, London School of Economics and Political Science, London, United Kingdom

The COVID-19 pandemic has been a global economic as well as health shock. Policy makers have had to make invidious choices in very uncertain circumstances, balancing strict public health measures to slow the spread of the virus against the adverse health, educational, and economic consequences of these choices. Attention has therefore rightly focused on the immediate mental health consequences of the pandemic, both for the general population and for people with pre-existing mental ill health, including impacts on rates of self-harm and completed suicide.

Evidence from a growing number of longitudinal studies indicates many sustained adverse impacts on mental health, albeit their magnitude varies across populations and countries. For instance, longitudinal analysis of 15,000 UK adults reported significantly increased levels of mental health problems for all age groups and genders, not only immediately after initial lockdown, but also in the subsequent months when restrictions were eased [1]. Effects appear especially pronounced for people with existing mental health problems, young people, women, and older people [2].

These adverse impacts on poor mental health did not always immediately translate into additional use of services. Early analyses suggest rates of hospital-presenting self-harm initially fell in countries hard hit during the first COVID wave, including England [3], France [4], and Spain [5]. Demand for mental health services is likely to have been suppressed, partially due to lockdown restrictions, as well as the understandable fear of contracting COVID in healthcare settings. In Ireland, for example, there is evidence that hospital presenting self-harm rates rose markedly after the initial fall at the beginning of the pandemic [6].

Much more will be learnt as longer-term data on mental health become available, including for individuals who have been bereaved or are living with long-COVID. However, policy makers and service planners cannot wait for long term impacts to become apparent, they need to formulate policy now to deal with the ongoing impacts of the pandemic and its aftermath over the next few years. It is therefore imperative that the mental health community is effective in its communications on the likely sustained adverse impacts on mental health of the pandemic.

To do this, we can draw on evidence from the past. Impacts on population mental health of previous economic shocks, including the 2008–2009 global economic crisis, have been analysed at length [7]. Most studies, across multiple countries, point to an association between economic shocks and enduring but time-delayed adverse impacts on population mental health, including suicidal behaviour. This is a critical message to convey to policy makers; even when the pandemic is fully contained there will be additional future demands on mental health services.

What distinguishes the current situation from previous crises has been the enormous and rapid investment in social protection measures in Europe to maintain the incomes of millions of people and stave off immediate unemployment and economic hardship. They have undoubtedly helped to protect mental health, but these welcome social protection measures are typically time limited. Any reduction in measures is likely to induce an economic shock and reveal what has hitherto been a hidden increase in unemployment. We also know from previous crises that involuntary shifts to part-time work, increased job insecurity and workplace downsizing, all of which may emerge, can be detrimental to mental health. At the same time demand for mental health services is likely to stay about normal levels, potentially for several years given the likely lagged effect between economic shocks and mental health impacts. For instance, in Sweden, suicide rates in men, and to a lesser extent women, who became long-term unemployed due to the economic crisis of the early 1990s increased significantly in the years after economic recovery began rather than during the crisis itself [8].

In all countries therefore, mid- to long-term policy plans to address the mental health consequences of the pandemic are needed. These plans need to be cross-sectoral, combining measures to protect and promote mental health with actions to treat additional mental ill-health. Of fundamental importance in all settings in Europe is the need for continued additional investment in income protection, bankruptcy and eviction protection measures that have been shown to protect mental health during and after an economic crisis [7,9]. If resources for these welfare measures are strained, they should be targeted at those economic sectors where the immediate risk of job-loss and unemployment post-pandemic are greatest.

Looking at some of the population groups whose mental health has been most disproportionately affected by the pandemic, children and young people would benefit from additional and sustained investment in online and face to face psychological counseling services. Health and social care workers and other frontline professionals who have experienced very high levels of psychological distress during the crisis also need support. Measures to protect their mental health need to be scaled up; these could perhaps include use of brief psychological interventions delivered by paraprofessionals for distress that have been used in conflict/disaster zones.

The pandemic has also changed the way in which mental health systems operate [10], with increased provision of online services and arguably a much greater focus on resilience and wellbeing. This has created great challenges, but it also has provided an opportunity to reshape mental health service delivery so that some of the new investments in online services continue to complement traditional mental health service models. This can help to maximise the opportunities to reach all individuals in mental distress. The temptation among some service planners to try and cut costs by overly relying on digital rather than face to face services should be resisted; such a move would potentially widen mental health inequalities between those who do and do not use digital technology.

Of course, long term mental health support plans need to be tailored to individual country contexts. The UK government’s recently published Covid-19 mental health and wellbeing recovery action plan for England provides a useful template for policy action [2]. This plan sets out multiple measures that are being taken within and beyond the health system. It includes actions targeted at children and young people, within and outside of school and higher education settings, including, for example, some additional support to help young people into employment training schemes. The plan also encourages frontline workers to make use of freely provided online psychological first aid training, both to improve their awareness of potential risks to mental health, as well as improving their understanding of when to signpost individuals toward specialist services. It recognises and describes specific mental wellbeing and loneliness alleviation initiatives to support other adversely affected groups including women, ethnic minorities, refugees, older people, the homeless, and prisoners.

The English plan also recognises the value of additional social protection measures and describes investment in the implementation of a debt respite scheme in which interest and charges on debts are frozen and enforcement action from creditors is paused for at least 60 days, alongside a package of other financial and regulatory measures to reduce risks to mental health that could arise from unmanageable debt. Within the health system, it sets out an aspiration for more integrated mental healthcare that is better linked to primary and community services, as well as sectors such as housing. It also commits to the continued option of face to face services, alongside expanded remote service provision.

While it is important for the mental health community to engage with policy makers on the shape of mid to long term mental health recovery plans, implementation will also require funding. As vaccination programmes are rolled out and infection rates fall, pressure will increase to reduce public expenditure. Historically, mental health services have been very vulnerable when public funding is constrained. After the 2008–2009 crisis, mental health systems in some European countries experienced deep budgetary cuts. That crisis economically is but a shadow of the current crisis, where astronomical levels of funds (mainly from borrowing) have been pumped into economies. The mental health community will have to make use of both health and economic arguments to highlight the potential adverse consequences of providing insufficient funding for implementation. The economic recovery in Europe depends on the physical and mental health of its citizens; support for mental health recovery needs to be accurately portrayed as a positive investment that will benefit society rather than a cost to be minimised.
